# Successful Treatment of Bladder Fungus Ball Due to *Candida auris* With Systemic/Local Amphotericin B and Surgical Excision

**DOI:** 10.1155/crpe/9741756

**Published:** 2025-05-11

**Authors:** Yağmur Erkol Yilmaz, Merve Havan, Eda Eyduran, Nilay Penezoğlu, Duygu Öcal, Serap Teber, Ergin Çiftçi, Yakup Tarkan Soygür, Tanıl Kendirli

**Affiliations:** ^1^Department of Pediatrics, Ankara University School of Medicine, Ankara, Türkiye; ^2^Department of Pediatrics, Division of Pediatric Intensive Care, Ankara University School of Medicine, Ankara, Türkiye; ^3^Department of Pediatrics, Division of Pediatric Infectious Disease, Ankara University School of Medicine, Ankara, Türkiye; ^4^Department of Medical Microbiology, Ankara University School of Medicine, Ankara, Türkiye; ^5^Department of Pediatrics, Division of Pediatric Neurology, Ankara University School of Medicine, Ankara, Türkiye; ^6^Department of Urology, Ankara University School of Medicine, Ankara, Türkiye

**Keywords:** *Candida auris*, children, fungus ball, urinary tract infection

## Abstract

Fungal infections in the urine are rare in healthy individuals but can occur in patients with diabetes, immunosuppression, urinary catheterization, prolonged hospital stays, and the use of broad-spectrum antibiotics. The most common strain is *Candida*, with *Candida albicans* being the most prevalent. *Candida auris* is a new emerged and severe, contagious species of *Candida* family especially in critically ill patients. We present a case of a 17-year-old male with neuromyelitis optica spectrum disorder who developed a fungus ball in the bladder. Despite multiple antibiotic therapies, persistent fevers led to the diagnosis of *Candida auris* and the detection of a fungus ball in the bladder. The patient was successfully treated with caspofungin, cystoscopy for total excision of the fungus ball, and intravesical liposomal amphotericin B. This case underscores the importance of early diagnosis and treatment of fungus balls to prevent complications such as obstructive complications and fungal urosepsis. In conclusion, identifying risk factors, such as immune dysregulation, prolonged PICU stay, mechanical ventilation, urinary catheter, and antibiotic use, is crucial in managing such cases.

## 1. Introduction

Although rare in the healthy population, fungal infections in the urine can be seen in patients with diabetes, immunosuppression, urinary catheterization, prolonged hospital stays, and the use of broad-spectrum antibiotics. The most frequently isolated strain is *Candida*, with *Candida albicans* (50%–70%), *Candida glabrata*, *Candida tropicalis*, and *Candida parapsilosis* being the most common species [1, 2]. As a rare complication, a fungus ball can form in the kidney, and rarely in the bladder, causing obstruction and requiring surgical excision. According to the literature, fungal pathogens most commonly affect the liver, spleen, and kidneys, with *Candida* spp. (*C. albicans*) being the most common agent, followed by *Aspergillus* spp. and *Mucor* spp [3]. Here, we present a case of a patient with seronegative neuromyelitis optica (NMO) spectrum disorder who developed a fungus ball in the bladder while being monitored in the pediatric intensive care unit (PICU).

## 2. Case Report

A 17-year-old male patient with no known prior illness was admitted with ascending muscle weakness starting from the lower extremities. Brain magnetic resonance imaging revealed T2 FLAIR hyperintense lesions in the subcortical and deep white matter of both cerebral hemispheres, corpus callosum, mesencephalon, and right pons, some of which showed contrast enhancement (MOGAD, NMO spectrum, and autoimmune encephalitis). Lumbar puncture tests showed no signs of infection, and immunoglobulins were negative. The patient was treated with 2 mg/kg/day intravenous immunoglobulin (IVIG) for 3 days and pulse steroids (30 mg/kg/day) for 5 days for autoimmune encephalitis. The patient was admitted to our PICU with a Glasgow Coma Scale score of 6, tetraplegia, and intubated. The patient received 7 sessions of plasmapheresis and 4 doses of rituximab (375 mg/m^2^/dose). Peripheral blood tests for antimyelin oligodendrocyte glycoprotein (MOG) antibody and aquaporin-4 antibody were negative. Electroencephalography was within normal limits. Due to the patient's inability to tolerate extubation, a tracheostomy was performed on the 38th day of hospitalization. Despite multiple antibiotic therapies, persistent fevers led to the initiation of fluconazole treatment for suspected fungal infection, which was used for 10 days. On the 39th day of monitoring, 100,000 CFU/mL of *Candida* colonies were isolated from the urine culture. The colonies were identified as *Candida auris* using MALDI-TOF Vitek-MS (bioMérieux, Marcy l'Etoile, France). Minimum inhibitory concentration (MIC) was performed using the sensititre yeast one (Thermo Fisher Scientific, USA). The MIC value for caspofungin was 0.25 μg/mL, for fluconazole was 32 μg/mL, for amphotericin was B 0.5 μg/mL, for micafungin ≤ was 0.06 μg/mL, and for anidulafungin was 0.12 μg/mL. The breakpoints were defined based on expert opinion published by the US Centers for Disease Control and Prevention (CDC) in 2017 and were amended in April 2019. Tentative MIC breakpoints of *Candida auris* by CDC are as follows: amphotericin *B* ≥ 2 μg/mL, anidulafungin ≥ 4 μg/mL, caspofungin ≥ 2 μg/mL, micafungin ≥ 4 μg/mL, fluconazole ≥ 32 μg/mL, and voriconazole and other second generation triazoles are not available [4].

**Figure 1:**
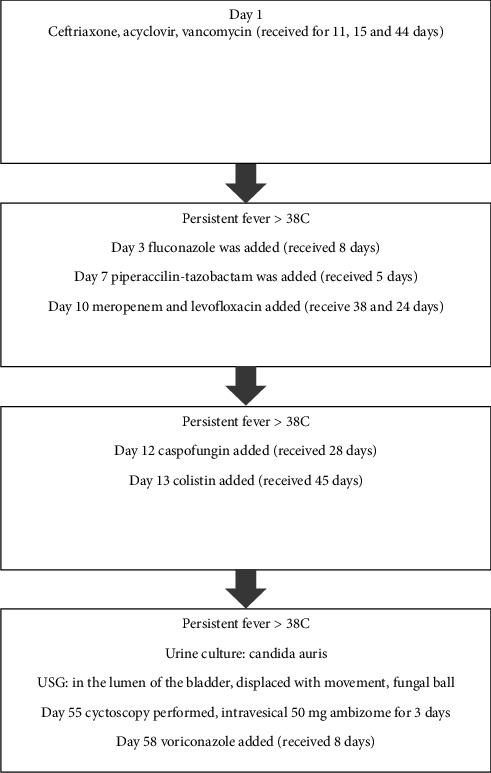
Treatment flow diagram of the patient.

The patient was rapidly isolated in accordance with infection controls. A body ultrasonography scan for additional fungal foci showed a heterogeneous echogenic area (fungal bezoar/fungus ball) in the bladder lumen, which moved with movement. Caspofungin was started on the 49th day of hospitalization and used for 21 days. Echocardiographic evaluation showed no infective endocarditis, and no ocular involvement was observed. On the 52nd day of monitoring, cystoscopy was performed, and a white, wall-adherent fungus ball was totally excised, and the intravesical tissue was completely cleaned; unfortunately, the tissue sample has not been histopathologically studied. On the 53rd day of hospitalization, *Candida auris* growth was reported in peripheral blood cultures at the 11th hour of incubation, MICs in increasing order were as follows: caspofungin 0.25 μg/mL, micafungin 0.12 μg/mL, amphotericin B 1 μg/mL, and fluconazole 256 μg/mL. Due to ongoing high fevers of the patient and newly discovered resistance to caspofungin, we decided to shift to amphotericin B. Intravesical liposomal amphotericin B treatment was also administered intravesical. The patient was monitored for 63 days in the PICU; he was administered wide spectrum antibiotics for a total of 56 days (piperacillin–tazobactam for 5 days, ceftriaxone for 11 days, levofloxacin 24 days, meropenem for 38 days, vancomycin for 44 days, and colistin for 45 days) and antifungals for 28 days (fluconazole for 8 days, caspofungin for 28 days, and voriconazole for 8 days) (Figure 1). He also received intravesical ambisome (50 mg) therapy for 3 consecutive days. Then, the patient was transferred to neurology ward and stayed for 56 days and 55 days in the physical therapy and rehabilitation unit, and he was discharged after a total of 6 months. Whole exome sequencing (WES) revealed a homozygous mutation in the CFI/NM_000204.5 gene (c.1a > G (pM1V) variant in the CFI gene). Complement Factor 1 (CF1) deficiency is an immune dysregulation that presents with recurrent pyogenic, skin, meningitis, respiratory, and urinary tract infections, as well as severe central nervous system inflammation (severe myelitis).

## 3. Discussion

Fungal detection in urinary tract infections is common. *Candida* species had seen in 4.3% of urine cultures in a referral children's hospital in a study. Candiduria was observed in patients with hospital stays longer than 1 month, use of at least two different antibiotics, ICU stays, and foreign body catheterization [5]. Retrospective analysis worldwide showed five most common factors found in patients were history of broad-spectrum antibiotic treatment (55.9%), central venous catheter (55.1%), intensive care unit (48.9%), urinary catheter (38.0%), and surgery (37.1%) [6]. Although generally asymptomatic, symptoms can be seen in immunocompromised patients and those with permanent bladder catheterization, and caution should be exercised regarding candidemia [1]. A multicenter study from Türkiye found that young age (1–24 months), infection with nonalbicans *Candida*, and the use of broad-spectrum antibiotics were risk factors for candidemia [7]. The Infectious Diseases Society of America recommends screening for fungal foci in the liver, spleen, and genitourinary system in patients with repeated *Candida* growth in blood cultures, but does not specify a preferred imaging method. The necessity and timing of imaging in invasive candidiasis remain controversial, but Sungkana et al. recommend imaging for pediatric patients at risk for fungal infections, including oncology patients, neonates, those on long-term broad-spectrum antibiotics, and immunosuppressed patients [3]. In a retrospective study by Devrim et al., 220 pediatric patients with candiduria were evaluated ultrasonographically, and renal fungus balls were detected in only 2 children (0.9%). However, the detection of underlying renal anomalies in 40.9% of the population suggests that evaluating organ involvement may be beneficial [8]. In critically ill patients with candiduria or those with impaired renal function, ultrasonography should be the first choice for imaging. In *Candida* pyelonephritis, focal, segmental, hypoechoic renal lesions may be seen on ultrasound, and fungus balls are also common in infants with candiduria in ICUs [9].

*Candida auris* has been a serious global health issue since it was first described in a case back in 2009, Japan. Notably, *Candida auris* is the first fungal pathogen to exhibit substantial and sometimes untreatable resistance to all major classes of antifungal drugs, including azoles, amphotericin B, and echinocandins and it has high mortality rates [10, 11]. Although it is rare, a total of 377 cases of *Candida auris* were presented in a review dated 2018, 17 of which were urinary tract infections [12]. In 2016, there were an outbreak occurred in Spain, where 140 patients were affected and today *Candida auris* is a well-known cause of nosocomial yeast infections worldwide [13]. In 2016 Centers for Disease Control and Prevention (CDC) published its report “Investigation of the First Seven Reported Cases of *Candida auris*, a Globally Emerging Invasive, Multidrug-Resistant Fungus” and stated that *Candida auris* can remain colonized on the skin of patients for a long time and grow in many samples taken from the patients' environment. Therefore, Standard and Contact Precautions for infection controls should be carefully followed [14].

A fungus ball in the bladder due to *Candida auris* is a very rare complication according to the literature, and risk factors in patients should be well identified. In our patient, the risk factors were immune dysregulation, prolonged PICU stay, prolonged mechanical ventilation support, urinary catheter use, and prolonged use of multiple antibiotics. While fluconazole is the first-choice drug, amphotericin B is also recommended for resistant groups. To prevent fungemia in *Candida*-associated fungus balls, guidelines recommend surgical excision with systemic antifungal treatment and/or local irrigation, as in our patient.

In conclusion, *Candida auris* has new emerged and easy contagious among critically ill patients, especially who has prolonged indwelled foreign healthcare tools such as CVC, urinary catheter. Early diagnosis and treatment of *Candida auris* associated fungus balls in the urinary system are important to prevent obstructive complications and fungal urosepsis. Surgical intervention and antifungal treatment (fluconazole, amphotericin B/flucytosine, and amphotericin B irrigation) are recommended for urinary tract infections complicated by fungus balls.

## Data Availability

The data supporting the findings of this case report are available within the article. Due to patient confidentiality, raw data (e.g., medical images and original medical records) are not publicly available.
